# Integration of Plant Defense Traits with Biological Control of Arthropod Pests: Challenges and Opportunities

**DOI:** 10.3389/fpls.2016.01794

**Published:** 2016-11-30

**Authors:** Julie A. Peterson, Paul J. Ode, Camila Oliveira-Hofman, James D. Harwood

**Affiliations:** ^1^Department of Entomology, West Central Research and Extension Center, University of Nebraska–Lincoln, North PlatteNE, USA; ^2^Department of Bioagricultural Sciences and Pest Management, Colorado State University, Fort CollinsCO, USA; ^3^Department of Entomology, University of Nebraska–Lincoln, LincolnNE, USA; ^4^Department of Entomology, University of Kentucky, LexingtonKY, USA

**Keywords:** host plant resistance, tritrophic interactions, transgenic crops, biological control, herbivore-induced plant volatiles (HIPVs)

## Abstract

Crop plants exhibit a wide diversity of defensive traits and strategies to protect themselves from damage by herbivorous pests and disease. These defensive traits may be naturally occurring or artificially selected through crop breeding, including introduction via genetic engineering. While these traits can have obvious and direct impacts on herbivorous pests, many have profound effects on higher trophic levels, including the natural enemies of herbivores. Multi-trophic effects of host plant resistance have the potential to influence, both positively and negatively, biological control. Plant defense traits can influence both the numerical and functional responses of natural enemies; these interactions can be semiochemically, plant toxin-, plant nutrient-, and/or physically mediated. Case studies involving predators, parasitoids, and pathogens of crop pests will be presented and discussed. These diverse groups of natural enemies may respond differently to crop plant traits based on their own unique biology and the ecological niches they fill. Genetically modified crop plants that have been engineered to express transgenic products affecting herbivorous pests are an additional consideration. For the most part, transgenic plant incorporated protectant (PIP) traits are compatible with biological control due to their selective toxicity to targeted pests and relatively low non-target impacts, although transgenic crops may have indirect effects on higher trophic levels and arthropod communities mediated by lower host or prey number and/or quality. Host plant resistance and biological control are two of the key pillars of integrated pest management; their potential interactions, whether they are synergistic, complementary, or disruptive, are key in understanding and achieving sustainable and effective pest management.

## Introduction to Key Concepts

The worldwide population is growing, with projections of 9–10 billion people living on Earth by 2050 (United Nations, 2004; Lutz and Samir, 2010). Global food demands are increasing concomitantly, with a need for heightened food security, increased agricultural productivity and improved water use efficiency of crops. In a global review of factors contributing to losses for eight major food and cash crops, animal pests came in second only to weeds, causing potential yield losses of 17.6% (Oerke and Dehne, 2004). Clearly, crop pests are responsible for significant losses to agricultural commodities worldwide despite profound efforts at management. Identification and promotion of sustainable solutions to these agricultural threats are essential for meeting future needs. The concepts of ***Integrated Pest Management*** (IPM), first championed by Stern et al. (1959), support practical efforts to achieve sustainable pest management. IPM has been described as “the harmonious use of multiple methods to control” pests, using “a set of decision rules based on ecological principles and economic and social considerations” (Kogan, 1998). Ideally, IPM incorporates the use of economic thresholds (Higley and Peterson, 2009) and a variety of control tactics (mechanical, physical, cultural, chemical, biological, and host plant resistance) making it essential to understand the interactions between different control tactics. Two key approaches for sustainable pest management have been (1) ***host plant resistance***, the selection or development (via traditional breeding or genetic modification) and use of crop plants that possess defensive traits against herbivores and disease, and (2) ***biological control***, the use of living organisms that are natural enemies of crop pests.

The concept of breeding plants to select for heritable traits that reduce pest impacts has been a part of agricultural production for over 100 years (Painter, 1951; Smith, 2005) and can be separated into tolerance and resistance mechanisms (Stout, 2013). ***Tolerance*** allows plants to withstand pest injury while ***resistance*** is conferred by plant traits that reduce the extent of pest injury and can be divided into constitutive or inducible and direct or indirect plant defenses (Stout, 2013). A ***constitutive defense*** is expressed in a plant regardless of whether it has been attacked by an herbivore, whereas an ***inducible defense*** is only expressed (or expressed to a greater degree) after attack. ***Direct defenses*** affect the herbivore without a mediating factor, whereas ***indirect defenses*** act via the actions of natural enemies. While indirect resistance may have the most obvious implications for biological control, other forms of resistance and tolerance also impact pest control by natural enemies. Holistic consideration of all these mechanisms is critical for their successful integration into pest control schemes.

Biological control programs use natural enemies (predators, parasitoids, and pathogens) of targeted pests to keep populations below the economic threshold. ***Classical biological control*** is the importation and establishment of natural enemies to control exotic pests while ***augmentation biological control*** incorporates the supplemental release of natural enemies. ***Conservation biological control*** involves modification of the environment or existing agronomic practices to protect and enhance specific natural enemies already present in the ecosystem (e.g., Landis et al., 2000; Eilenberg et al., 2001). The maintenance of natural enemy populations via conservation biological control can be a practical and sustainable option for low-value and high-acreage commodities, such as maize and other annual field crops (Thorbek et al., 2004; Naranjo et al., 2015). The responses of natural enemies to pest population changes are critically important and these can be classified as ***numerical*** (changes in natural enemy abundance due to reproduction or aggregation) or ***functional*** (changes in natural enemy behavior) (Hajek, 2004). Seminal work on functional responses of predators to their prey items by Holling (1966) demonstrated that rate of prey discovery, search time, handling time, and predator hunger were important factors in determining functional response. In the years since Holling’s research, studies in pest management have frequently examined how predators respond to prey, documenting the existence of functional responses in the context of biological control (e.g., De Clercq et al., 2000; Lee and Kang, 2004; Rutledge and O’Neil, 2005). Interestingly, some studies also describe variable responses of predators on different plants using plant-based defenses such as glandular trichomes and allelochemical production (De Clercq et al., 2000). These variable responses therefore highlight the need for careful consideration of the effects of different plant traits on pest suppression.

The interactions between plants, herbivores, and their natural enemies are referred to as ***tritrophic interactions*** and this multi-trophic exchange is key to understanding the interactions between host plant resistance and biological control. Natural enemies can be considered an extension of plant defense if plant traits, such as release of herbivore-induced plant volatiles (HIPVs), draw in these natural enemies. The literature is replete with examples of natural enemies acting in a top-down fashion, reducing herbivore populations, thereby providing plant defense.

The intention of this section is to provide a general introduction to the key concepts that provide context for the remainder of this review article. For more in-depth discussion of these topics, please refer to the many texts that review these topics (i.e., Painter, 1951; Panda and Khush, 1995; Kogan, 1998; Bellows et al., 1999; Agrawal, 2000a; Landis et al., 2000; Hajek, 2004; Smith, 2005; Heil, 2008; Radcliffe et al., 2009; van Lenteren, 2012; Stout, 2013; Pedigo and Rice, 2014). This review will focus on the interactions between biological control and host plant resistance, addressing the mechanisms and potential outcomes of interactions, with special attention to genetically modified insect-resistant crops and case studies for application of host plant resistance and biological control in cropping systems.

## Impact of Plant Traits on Biological Control

The mechanisms by which plant defensive traits can affect biological control can be divided into four major categories: semiochemically, plant toxin-, plant nutrient-, and physically mediated interactions. These have been widely recognized as the major mechanisms by which the three trophic levels interact (Price, 1986; Thomas and Waage, 1996; Agrawal, 2000a) and will be reviewed in detail here. Their integration (see Discussion) into biological control programs is critical as we develop sustainable solutions for pest management.

### Semiochemically Mediated Interactions

Plants produce a wide range of volatile compounds that are the predominant signals used by arthropod herbivores to locate suitable host plants (Schoonhoven et al., 2005). These volatile profiles can change both quantitatively and qualitatively following herbivory (Dicke, 1999; Páre and Tumlinson, 1999; Heil and Ton, 2008), dramatically altering their attractiveness (or repellency) to herbivores and their natural enemies (Heil, 2014). Feeding, especially by chewing herbivores, results in mechanical damage to plant tissues eliciting a wound response thereby creating electrical, hydraulic, and chemical signals (e.g., systemin; Kessler and Baldwin, 2002). This action results in local and systemic release of linolenic acid from plant cell membranes and is converted by the enzyme lipoxygenase (LOX) to 13-hydroperoxide, which enters one of two pathways (Walling, 2000; Kessler and Baldwin, 2002). In one pathway, 13-hydroperoxide may be hydrolyzed by hydroperoxide lyase to yield ‘green leaf volatiles’ (GLVs; e.g., C_6_ alcohols and aldehydes) and these, and other volatiles such as terpenoids, are often considered indirect defenses because they attract natural enemies. Alternatively, 13-hydroperoxide can enter the octadecanoid pathway, resulting in the production of jasmonic acid (JA), ultimately producing an array of anti-herbivore defenses including proteinase inhibitors (anti-digestive proteins), polyphenol oxidases (anti-nutritive enzymes), and a bewildering diversity of plant-specific toxins (Walling, 2000; Kessler, 2015; see Plant Toxin-Mediated Interactions). These inducible defensive chemicals are generally termed direct defenses in that they directly deter or inhibit feeding by herbivores.

Yet, plant responses to herbivory are more complex than simple wound responses to mechanical damage, which cannot explain the specificity of some plant responses to herbivores. In addition to physical damage, herbivores secrete substances that may modify plant responses. Collectively, these substances are referred to as herbivore-associated molecular patterns (HAMPs; Felton and Tumlinson, 2008; Mithöfer and Boland, 2008) and include substances such as regurgitants and salivary secretions (Alborn et al., 1997; Musser et al., 2002; Schäfer et al., 2011; Tian et al., 2012; Louis et al., 2013), and even frass production (Ray et al., 2015). Behavioral interactions, too, modify plant volatile production with walking on leaf surfaces (Tooker et al., 2010) and oviposition (Hilker and Meiners, 2006; Kim et al., 2012; Hilfiker et al., 2014) having profound effects. It is therefore unsurprising that plants respond to herbivory in specific ways that provide informative semiochemical-based information for both herbivores and their natural enemies. Plants emit different suites of volatiles, attracting different parasitoid complexes, depending on the species of herbivore attacking the plant. Clearly, there is abundant evidence that HAMPs and behavioral interactions of herbivores with host plants alter plant defensive responses beyond that of simple mechanical damage (e.g., Dicke, 1999; Reymond et al., 2000; Kessler and Baldwin, 2002). This highlights a cautionary note when interpreting findings of the large number of ecological studies using artificial leaf clippings and hole punches as a proxy for herbivore damage.

As discussed above, plant volatiles that attract natural enemies are considered indirect defenses (Vet and Dicke, 1992; Kessler and Baldwin, 2002; Turlings and Wäckers, 2004; Wäschke et al., 2013). These GLVs, and others produced via different pathways such as volatile terpenoids (Kessler and Baldwin, 2002; Dudareva et al., 2013; Kessler, 2015), play a crucial role in signaling specific information for parasitoids regarding the status of herbivores and their natural enemies. The information conveyed in HIPVs can provide information on the species of herbivore present, the level of herbivory damage sustained, the developmental stage of the host, and even whether the herbivore has been previously parasitized. For instance, tomato plants attacked by tobacco budworm *Heliothis virescens* (F.) (Lepidoptera: Noctuidae), but not the closely related tomato fruitworm *Helicoverpa zea* (Boddie) (Lepidoptera: Noctuidae), emit a volatile profile that is highly attractive to the specialist parasitoid of the tobacco budworm, *Cardiochiles nigriceps* Viereck (Hymenoptera: Braconidae) (De Moraes et al., 1998). Such information conveyed to natural enemies has profound consequences for the biological control services afforded by them and maximizes the top-down effect of such species on herbivorous pests. The quantity of HIPVs released may reflect the level of herbivory and determine the level of attractiveness to parasitoids. In studies of *Cotesia glomerata* (L.) (Hymenoptera: Braconidae) attacking *Pieris rapae* (L.) (Lepidoptera: Pieridae), plants attacked by more herbivores or induced with higher concentrations of JA (simulating higher levels of herbivory) were more attractive to *C. glomerata* (Geervliet et al., 1998; Bruinsma et al., 2009). Yet, HIPV production may also influence the plant’s attractiveness to herbivores. In an interesting study of two chrysomelid beetles (*Gynandrobrotica guerreroensis* (Jacoby) and *Cerotoma ruficornis* Olivier) attacking wild lima beans [*Phaseolus lunatus* L. (Fabales: Fabaceae)], female beetles were repelled by HIPVs produced by induced plants regardless of level of induction (possibly reflecting competition and a lack of enemy-free space) whereas males were attracted by weakly induced plants (possibly indicating the presence of a mate) but repelled by strongly induced plants (Ballhorn et al., 2013). The effect of such changes in herbivore densities on parasitoid foraging decisions is unexplored. Furthermore, parasitoid species identity may also influence plant volatile production. Cabbage [*Brassica oleracea* L. (Brassicales: Brassicaceae)] produced similar HIPV profiles when attacked by imported cabbageworm *Pieris rapae* (L.) or large cabbage white *P. brassicae* (L.) (Lepidoptera: Pieridae) (Poelman et al., 2011). Yet, intriguingly, herbivore regurgitant characteristics were strongly influenced by the species of parasitoid developing within the herbivore, which differentially expressed genes within the plant’s JA-signaling pathway. Even hyperparasitoids use HIPVs to locate their parasitoid hosts; the hyperparasitoid *Lysibia nana* Gravenhorst (Hymenoptera: Ichneumonidae) was more attracted to *P. rapae* hosts attacked by *C. glomerata* than those attacked by *C. rubecula* or unparasitized hosts. Field surveys showed hosts parasitized by *C. glomerata* are more likely to be hyperparasitized than *C. rubecula*-parasitized hosts and this preference was due to differences in HIPV profiles elicited by the oral secretions of *P. rapae* (Poelman et al., 2012). The sheer complexity of such semiochemically mediated interactions demonstrates the need for consideration of the multitude of factors influencing pest control, rather than single elements acting along.

#### Case study: Maize Volatiles, Western Corn Rootworm, and Entomopathogenic Nematodes

Domestication can inadvertently alter the volatile profiles of many crop plants, affecting rates of parasitism. One example is the production of the sesquiterpene (E)-β-caryophyllene (EβC) in maize. EβC is emitted in response to above- (Turlings et al., 1998) and below-ground injury (Rasmann et al., 2005). It serves as an attractant for natural enemies of maize pests (Rasmann et al., 2005; Köllner et al., 2008) and provides protection from herbivores with different modes and sites of attack (Köllner et al., 2008). Unfortunately, EβC production has been unintentionally bred out of commercially available North American maize hybrids, but it is still present in European maize lines and teosinte (*Zea mays* ssp. *parviglumis*) (Degen et al., 2004; Rasmann et al., 2005). EβC production can be reintroduced by insertion of a gene from oregano, *Origanum vulgare* L. (Lamiales: Lamiaceae) (Degenhardt et al., 2009), demonstrating the ability to genetically enhance crops to increase natural enemy control of insect pests.

The most challenging belowground pest of maize production in North America and Europe is the western corn rootworm (WCR) *Diabrotica virgifera virgifera* LeConte (Coleoptera: Chrysomelidae). Upon injury to the roots, European maize hybrids induce a strong production of EβC locally and a weak systemic response throughout root tissues (Hiltpold et al., 2011). EβC released into the rhizosphere recruits the entomopathogenic nematode (EPN) *Heterorhabditis megidis* Poinar, Jackson and Klein (Rhabditida: Heterorhabditidae). In field studies, maize hybrids producing EβC had significantly higher rates of *H. megidis* infection in WCR larvae and reduced rootworm adult emergence than non-EβC-emitting hybrids; non-EβC-emitting maize varieties do not recruit *H. megidis* when attacked by the WCR (Rasmann et al., 2005).

Numerous studies have shown the potential of EPNs to suppress WCR populations (Wright et al., 1993; Jackson, 1996; Toepfer et al., 2005, 2008; Kurtz et al., 2009; Hiltpold et al., 2012) but not all EPN species and strains that attack WCR larvae are attracted to EβC (Hiltpold et al., 2010c; Anbesse and Ehlers, 2013; Laznik and Trdan, 2013). *Heterorhabditis bacteriophora* Poinar (Rhabditida: Heterorhabditidae), for instance, is highly effective against WCR larvae (Jackson, 1996; Toepfer et al., 2008; Pilz et al., 2009) but is not attracted to EβC (Hiltpold et al., 2010a,c). Selective breeding of *H. bacteriophora*, however, can increase the attraction of infective juveniles to EβC-emitting maize roots, thereby increasing WCR mortality (Hiltpold et al., 2010a,b).

Maximizing the expression of HIPVs via bioengineering, while increasing EPN responsiveness to volatiles, can help enhance the effectiveness of biological control in crops. However, more studies are needed to assess the costs, viability and potential risks of introducing EβC-emitting maize varieties with EPN releases. The WCR has a high propensity for invasion and adaptation (Gray et al., 2009) and has already developed resistance to multiple chemical (Meinke et al., 1998; Ciosi et al., 2009; Pereira et al., 2015), genetic (Gassmann et al., 2011; Wangila et al., 2015), and cultural (Levine et al., 2002) management tools. Alternative control strategies, such as recruitment of entomopathogens using plant volatiles, must be explored in order to sustainably manage this critical pest.

### Plant Toxin-Mediated Interactions

Of the more than 100,000 identified plant secondary metabolites, many play roles in direct defense against herbivorous insects through anti-nutritive, anti-digestive, or toxic compounds. Many of these defensive chemicals are produced constitutively, regardless of whether a plant is attacked by herbivores; others are often inducible via the JA-based signaling pathway described in Semiochemically Mediated Interactions above (Memelink et al., 2001; Agrawal, 2011; De Geyter et al., 2012). While plant anti-herbivore toxins might be expected to exhibit similar responsiveness as semiochemicals to the damage done by specific herbivores and the presence of their natural enemies, little evidence suggests this is the case. Rather, many secondary compounds are present within only a limited range of plant families (e.g., the glucosinolates are found almost exclusively in plants in the Order Brassicales (Halkier and Gershenzon, 2006), furanocoumarins are primarily associated with the families Apiaceae and Rutaceae (Berenbaum, 1983, 1990)). Specificity of plant defensive responses to different herbivores (‘specificity of elicitation’ *sensu* Stout et al., 1998) seems, for the most part, to be quantitative rather than qualitative. For instance, levels of damage caused by different herbivores (Van Zandt and Agrawal, 2004) or variable damage by unparasitized vs. parasitized herbivores that results in differential feeding by herbivores (Ode et al., 2016) may result in the induction of different plant defensive compounds. While some evidence indicates that different herbivores can differentially induce plant defenses (e.g., Stout et al., 1998; Agrawal, 2000b; Poelman et al., 2008), the effects on higher trophic levels are poorly studied.

Unlike indirect defenses (see Semiochemically Mediated Interactions), direct plant defenses typically have negative effects on parasitoid fitness (Ode, 2006, 2013) and occur through one of three, non-mutually exclusive routes. Plant toxins may: (1) reduce host size, having negative consequences for parasitoids feeding on such hosts, (2) pass unmetabolized through the herbivore’s midgut into the hemolymph where they are directly encountered by developing parasitoid larvae (Campbell and Duffey, 1979; McGovern et al., 2006; Lampert et al., 2008), or (3) be sequestered for defense against their own natural enemies (Nishida, 2002; Ode, 2006; Lampert et al., 2011a). For example, the catalpa sphinx moth, *Ceratomia catalpae* (Boisduval) (Lepidoptera: Sphingidae), sequesters the iridoid glycoside catalpol when it feeds on the catalpa plant, *Catalpa bignonioides* Walter (Lamiales: Bignoniaceae) (Lampert et al., 2010). Interestingly, the parasitoid *Cotesia congregata* (Say) (Hymenoptera: Braconidae) appears to be little affected by concentrations of catalpol, which also accumulate in the tissues of the parasitoid suggesting the role of this compound as protection against its own hyperparasitoids (Lampert et al., 2011a).

Whether parasitoids are adversely affected by plant toxins depends in large part on the level of host plant specialization of their herbivorous hosts. The diversity of host plants on which a given herbivore develops depends, in part, on its ability to metabolize or avoid plant defensive toxins (Schoonhoven et al., 2005). Herbivores feeding on a broader range of host plants typically possess detoxification enzyme systems capable of metabolizing a broad array of plant toxins (Krieger et al., 1971; Li et al., 2004; Ali and Agrawal, 2012). Conversely, herbivores with specialized diets tend to have more efficient detoxification enzymes that metabolize the narrower range of plant toxins to which they are exposed (Wittstock et al., 2004; Mao et al., 2006). Far less documentation exists regarding the consequences for parasitoids of developing in generalist vs. specialist herbivores because few studies have documented the levels of unmetabolized plant toxins in the hemolymph of herbivores with different diet breadths. In one study, significantly more xanthotoxin was passed unmetabolized into the hemolymph of the cabbage looper, *Trichoplusia ni* (Hübner) (Lepidoptera: Noctuidae), a generalist herbivore, than was passed in the hemolymph of the parsnip specialist *Depressaria pastinacella* (Geeze) (Lepidoptera: Oecophoridae) (Lampert et al., 2011b). In turn, *Copidosoma floridanum* Ashmead (Hymenoptera: Encyrtidae) (a parasitoid of *T. ni*) suffered increased mortality and reduced clutch sizes relative to *Copidosoma sosares* (Walker) (Hymenoptera: Encyrtidae) (a specialist parasitoid of *D. pastinacella*) even though both herbivore-parasitoid combinations were reared on the same artificial diets (Lampert et al., 2011b). Other studies have documented similar patterns (e.g., Barbosa et al., 1986, 1991). Finally, generalist and specialist herbivores of cruciferous plants are negatively affected by different classes of glucosinolates. Generalist herbivores are typically susceptible to both indole and aliphatic glucosinolates, whereas specialist herbivores are susceptible to just indole glucosinolates (Gols et al., 2008a,b; Müller et al., 2010; Harvey and Gols, 2011). However, some specialists are known to sequester glucosinolates, providing protection against their natural enemies [e.g., the turnip sawfly *Athalia rosae* (Hymenoptera: Tenthredinidae) (Müller et al., 2002) and the specialist aphids *Brevicoryne brassicae* (L.) and *Lipaphis erysimi* Kaltenbach (Hemiptera: Aphididae)] (Francis et al., 2001; Rossiter et al., 2003; Kazana et al., 2007). Interestingly, survivorship and body size of unparasitized *T. ni* were negatively correlated with concentrations of aliphatic glucosinolates whereas survivorship and clutch sizes of *T. ni* parasitized by *C. floridanum* were negatively affected by concentrations of indole (and not aliphatic) glucosinolates (Ode et al., 2016).

Despite long-running discussions about the potential (in)compatibilities of biological control and breeding programs for plant resistance (e.g., Bergman and Tingey, 1979; van Emden, 1991; Bottrell et al., 1998; Cortesero et al., 2000; Poppy and Sutherland, 2004), surprisingly little is known about the severity of these incompatibilities. This is primarily a reflection of the independent paths that host plant resistance and biological control programs have taken; i.e., IPM is rarely practiced in reality. Part of the difficulty lies in the fact that when crop varieties are bred for insect resistance, rarely do we know the exact mechanism involved. Nonetheless, breeding programs likely select for plant defensive toxins in many cases, which likely mediate resistance. When true, we expect that many of the patterns outlined above will hold. For instance, soybeans, *Glycine max* (L.) (Fabales: Fabaceae), with the *Rag1* gene are resistant to soybean aphid *Aphis glycines* Matsumura (Hemiptera: Aphididae). Compatibility studies between *Rag1* and biological control agents of *A. glycines* have shown that these agents are less effective (e.g., reduced foraging efficiency and survivorship) on soybean varieties containing the resistant *Rag1* gene (Lundgren et al., 2009b; Ghising et al., 2012; Ode and Crompton, 2013).

#### Case Study: Cotton, Gossypol and Bt Toxins, Herbivores, and Natural Enemies

Cotton, *Gossypium hirsutum* L. (Malvales: Malvaceae), the most important plant-based fiber used by humans worldwide, presents an interesting example of the difficulties in breeding for resistance against multiple insect pests. It is consumed by a large number of insect herbivores including the boll weevil, bollworm, pink bollworm, tobacco budworm, armyworms, cotton aphid, whiteflies, *Lygus* bugs, and thrips (Matthews and Tunstall, 1994; Hagenbucher et al., 2013a). Prior to the introduction of *Bacillus thuringiensis* (Bt) cotton and more effective IPM approaches, insecticides were the primary means of pest control. An array of morphological (e.g., trichomes) and chemical defenses are produced by cotton and of the chemical defenses, terpenoids (especially gossypol and related compounds) are the best studied. Gossypol, present in leaves and seeds, provides resistance to a broad range of lepidopteran pests (Bottger and Patana, 1966). As it is also toxic to humans, breeding efforts have selected for glandless cultivars that produce low gossypol levels, but these cultivars are particularly susceptible to a range of insect pests (Jenkins et al., 1966). Recent efforts using RNAi to produce low gossypol levels in the seeds while maintaining high levels elsewhere have been successful (reviewed in Hagenbucher et al., 2013a), but gossypol also has negative effects on some natural enemies. For instance, *Campoletis sonorensis* (Cameron) (Hymenoptera: Ichneumonidae) experiences reduced body size, reduced survivorship, and increased development time when developing on *H. virescens* that had fed on diets high in gossypol (Gunasena et al., 1989), although this negative effect is by no means universal across species (e.g., Sun et al., 2011). Similar to semiochemically induced effects, responses of organisms to different compounds are specific to the exact plant–insect interaction.

The recent focus in cotton breeding for insect herbivore resistance has centered on the development of Bt transgenic lines expressing Cry-endotoxins that confer resistance against lepidopteran herbivores. In particular, adoption of Bt cotton has been credited with the eradication of the pink bollworm *Pectinophora gossypiella* (Saunders) (Lepidoptera: Gelechiidae) in the southwestern United States (Carriére et al., 2003) and substantial declines of *Helicoverpa armigera* (Hübner) (Lepidoptera: Noctuidae) in China (Wu et al., 2008). The specificity of Cry toxins against lepidopterans and reduced pesticide use after widespread adoption of Bt cotton has provided an environment favorable to natural enemies, allowing increased control of a wide variety of cotton pests (Naranjo, 2011; Lu et al., 2012). However, Bt has not been without its downsides as damage by some pests, for example, mirid bugs (Lu et al., 2010), have been documented to increase with the widespread use of Bt cotton, presumably because of competitive release from lepidopterans. Another complication involves improved success of the cotton aphid *Aphis gossypii* Glover (Hemiptera: Aphididae) on Bt cotton. Suppression of feeding by lepidopteran herbivores on Bt cotton reduces induction of key defensive terpenoids, such as gossypol, making these plants much more susceptible to aphids, which do not induce terpenoids (Hagenbucher et al., 2013b). Furthermore, induced terpenoids from non-Bt cotton end up in the hemolymph of the aphids, reducing success of attack by the parasitoid *Lysiphlebus testaceipes* (Cression) (Hymenoptera: Braconidae) (Hagenbucher et al., 2014b). Reduced parasitism was most likely due to reduced parasitoid acceptance of aphids feeding on lepidopteran-infested non-Bt cotton. Finally, as honeydew is an important source of nutrition for foraging parasitoids, the effect of honeydew from lepidopteran-infested Bt and non-Bt cotton on two important parasitoids of cotton pests, *L. testaceipes* and the whitefly parasitoid *Eretmocerus eremicus* Rose and Zolnerowich (Hymenoptera: Aphelinidae) was compared. While gossypol and other terpenoids were significantly higher in the honeydew produced on lepidopteran-infested non-Bt cotton, this did not affect the quality of the honeydew in terms of its effects on parasitoid longevity or fecundity (Hagenbucher et al., 2014a).

### Plant Nutrient-Mediated Interactions

The proteins, sugars, lipids, nucleic acids, vitamins, and minerals contained within plant tissue provide the nutrition necessary for growth, development, and survival of many insects. In turn, the nutrients provided by plants to herbivores affect the nutrients subsequently available to their natural enemies. The presence, quantity, quality, and availability of these nutrients varies significantly between plant species and varieties, and can be affected by season, plant phenology, and other biotic and abiotic conditions (Fox et al., 1990; Roth and Lindroth, 1995; Walde, 1995; Stadler and Mackauer, 1996).

A key indirect interaction between host plant nutrition and natural enemies occurs when herbivore growth and development is delayed by suboptimal plant quality, extending the period of time when herbivores are vulnerable to attack (Moran and Hamilton, 1980; Price et al., 1980; Price, 1986; Loader and Damman, 1991; reviewed in Benrey and Denno, 1997). An example of this “slow-growth–high-mortality” hypothesis was reported for the Mexican bean beetle *Epilachna varivestis* Mulsant (Coleoptera: Coccinellidae) feeding on soybean. The spined soldier bug, *Podisus maculiventris* (Say) (Hemiptera: Pentatomidae), was better able to control *E. varivestis* on crop varieties that lowered the herbivore’s growth rate (Price et al., 1980), although the exact resistance mechanism was not known. In addition to a longer period of vulnerability, a slow herbivore growth rate can be advantageous if the natural enemy’s functional response is stronger when consuming smaller prey, as tends to be the case with predators (Price, 1986). Insect pathogens, in particular, are positively associated with the slow-growth–high-mortality hypothesis (Schuster et al., 1983; Hamm and Wiseman, 1986). In one case, *S. frugiperda* feeding on resistant maize plants had reduced growth and vigor, making them more susceptible to infection with nuclear polyhedrosis virus (NPV) (Hamm and Wiseman, 1986). However, the slow-growth–high-mortality hypothesis does not hold true for all tritrophic interactions. For example, Leather and Walsh (1993) found that pine beauty moth *Panolis flammea* Denis and Schiffermüller (Lepidoptera: Noctuidae) larvae were not more vulnerable to natural enemies when development was delayed by host plant quality. Some natural enemies, such as parasitoids, may actually be at a disadvantage when their hosts are smaller and/or of lower quality, and smaller hosts may also affect the sex ratio and fecundity of parasitoid populations (Kuo, 1986). It is therefore important to examine whether the presence of smaller and lower quality hosts due to suboptimal plant nutrition has a large enough impact on parasitoids as to affect their ability to suppress pest populations.

Many natural enemies also engage in omnivory, supplementing their prey-based diet with plant-provided resources (reviewed in Lundgren, 2009), particularly during periods when prey abundance is low. This can allow for more stable interactions between predators and prey (Agrawal, 2000a) and may facilitate early season colonization of crop fields and better pest suppression due to this “lying in wait” of natural enemies prior to arrival of the pest species (Settle et al., 1996; Eubanks and Denno, 1999; Athey et al., 2016). Therefore, good quality plant hosts in the case of omnivorous natural enemies is essential for a positive relationship between plant and biocontrol. Plants expressing herbivore defense traits can have direct impacts on facultatively phytophagous predators but the literature is lacking in how these interactions will impact the compatibility of host plant resistance with biological control (Lundgren, 2009).

Some insects are truly omnivorous, having a flexible trophic strategy that allows them to utilize either plant or prey resources, with the potential to inflict crop damage if engaging in phytophagy. For example, the western flower thrips *Frankliniella occidentalis* (Pergande) (Thysanoptera: Thripidae) feeds on plant material and arthropod prey, leading to its role as both a serious pest (Grazia-Tommasini, 1995; Kirk and Terry, 2003) and a biological control agent (Trichilo and Leigh, 1986; Wilson et al., 1996; Agrawal and Karban, 1997; Milne and Walter, 1997). Furthermore, Agrawal et al. (1999) revealed that the presence of prey [eggs of the Pacific spider mite *Tetranychus pacificus* McGregor (Thysanoptera: Tetranychidae)] reduced feeding by *F. occidentalis* on cotton by nearly 50%. However, when cotton plants were first exposed to feeding pressure by spider mites, eliciting systemically induced plant defenses that lower host plant quality, herbivory by *F. occidentalis* was reduced (Agrawal et al., 1999). When both induced host plant defenses and *T. pacificus* egg prey were available, feeding preference shifted to consume half the amount of cotton tissue and twice the number of prey (Agrawal et al., 1999). Thus, host plant quality and prey availability are important factors for arthropods with omnivorous trophic tendencies.

Extrafloral nectaries (EFN) are a plant-provided resource that deserve additional attention because of their role in natural enemy nutrition. It is hypothesized that the main function of extrafloral nectar is to recruit predators and parasitoids for the protection of the plant against herbivores, an example of indirect host plant resistance (Bentley, 1977; Koptur, 1992; Turlings and Wäckers, 2004). Some EFN emit olfactory signals that are attractive to natural enemies, such as parasitoids (Lewis and Takasu, 1990; Stapel et al., 1997). By providing nutritional resources, the presence of EFN can lead to enhanced herbivore suppression by arthropod natural enemies, such as ants (Bentley, 1977; Smiley, 1986), spiders (Ruhren and Handel, 1999), predatory mites (Bakker and Klein, 1992), coccinellids (Stephenson, 1982) and parasitoids (Lindgren and Lukefahr, 1977). Interestingly, some plants produce a consistent low level of EFN, but increase production in response to herbivory; in this manner, extrafloral nectaries can be considered both constitutive and inducible indirect host plant resistance (Wäckers et al., 2001; Wäckers and Bonifay, 2004; Lundgren, 2009; Heil, 2015). The applied implications of EFN production by crop plants is examined in the case study with cotton below.

#### Case Study: Extrafloral Nectar-Producing Cotton, Its Herbivores, and Natural Enemies

The ability of extrafloral nectar to attract natural enemies for biological control of cotton pests has long been exploited. Cook (1904, 1905) reported on the practice of indigenous farmers in Guatemala, who purposely cultivated cotton near nests of the tropical ant *Ectatomma tuberculatum* (Olivier) (Hymenoptera: Formicidae). In addition to feeding on EFN, these ants attacked boll weevil *Anthonomus grandis* Boheman (Coleoptera: Curculionidae) adults. Subsequently, plant breeding efforts in the mid 1900’s attempted to develop cotton varieties that lacked EFN, due to the observation that both natural enemies and some lepidopteran pests, such as *P. gossypiella*, benefitted from cotton nectaries (Lukefahr and Griffin, 1956; Lukefahr and Rhyne, 1960; Bentley, 1983). However, the benefit of a modest reduction in lepidopteran pests was outweighed by the disadvantage of reduced natural enemy populations, although this conclusion was doubted at the time (Rogers, 1985; Schuster and Calderon, 1986). The population of natural enemies in “nectarless” cotton varieties was up to 35% lower than EFN-producing cotton and the presence of EFN in cotton had positive impacts on the attraction, retention, and efficiency of many predators, including chrysopids, anthocorids, and coccinellids (Schuster et al., 1976). Similarly, the parasitoid *Microplitis croceipes* (Cresson) (Hymenoptera: Braconidae), which attacks larvae of the bollworm *H. zea*, is stimulated to stay longer and attack a greater number of hosts in the presence of nectar (Stapel et al., 1997). Many other examples exist in the literature, providing clear evidence for widespread benefits of EFN to parasitoids (e.g., Treacy et al., 1987). Another functional group of natural enemies, cursorial wandering spiders such as *Cheiracanthium inclusum* (Hentz) (Araneae: Miturgidae) and *Hibana futilis* (Banks) (Araneae: Anyphaenidae), are important nocturnal predators of lepidopterous pest eggs in cotton (Pfannenstiel, 2008) and consume EFN in the field (Taylor and Pfannenstiel, 2008). Furthermore, *Hibana futilis* responds to olfactory cues from extrafloral nectar and engages in restricted area searching following contact with nectar (Patt and Pfannenstiel, 2008, 2009) and profound improvements of survival are evident when provided EFN in the diet (Taylor and Pfannenstiel, 2009; Pfannenstiel and Patt, 2012).

The majority of modern cotton varieties now produce EFN, but past breeding efforts illustrate the difficulty in managing plant traits affecting both pests and natural enemies. Rogers (1985) recommended that for the case of nectar-producing cotton, varieties should be developed that produce nectar that is palatable to beneficial species, but not pests. However, the feasibility of this suggestion has not been explored. Recommendations to improve the recruitment of natural enemies to cotton fields include selecting for varieties with enhanced nectar production. For example, most cotton leaves bear a single nectary, but some have three (Cortesero et al., 2000) and a breeding challenge is whether cotton varieties can be developed with a greater number of nectaries. It is evident that plant nutrients are critically important to a diverse array of natural enemies across multiple functional groups. Integration of this resource into biological control programs through selective enhancement or provisioning of additional nectar sources can assist when developing sustainable solutions to pest management. Clearly, challenges exist when selectively breeding for plant defense traits (described here and in other sections), but careful consideration of their integration with biological control can provide synergistic levels of pest control.

### Physically Mediated Interactions

Just as some tritrophic interactions involve both semiochemicals and toxins, physically mediated interactions do not always function alone. For example, substances such as resin or latex physically limit herbivores by trapping or immobilizing them, while simultaneously delivering various toxins (Konno, 2011), and glandular trichomes release sticky and toxic compounds serving as a physical and chemical defense against herbivores (Levin, 1973; Southwood, 1986; Cortesero et al., 2000).

Plant architecture affects the dispersion of herbivores on a host plant, which may in turn affect searching behavior and host-finding abilities of natural enemies. For example, the leaves of winter wheat varieties developed for resistance to Russian wheat aphid *Diuraphis noxia* (Kurdjumov) (Hemiptera: Aphididae) remain flat, compared to susceptible varieties whose leaves furl in response to aphid feeding (Hawley et al., 2003), exposing aphids to disturbances such as wind, rain, and predators inducing them to fall from the plant (von Berg et al., 2008). Characteristics that affect falling behavior of herbivores can affect predation rates as they experience vulnerability to ground-dwelling predators and may also face additional challenges from natural enemies as they attempt to recolonize the plant (Sunderland et al., 1986; Winder, 1990; Winder et al., 1994).

The size and morphology of certain plant structures that confer resistance to herbivores can affect biological control by altering where pests feed, how long they are exposed and how apparent or accessible the pests are to natural enemies, particularly if plant morphology can delay internally feeding pests from entering the plant’s tissues. An example would be husk tightness and length in sweet corn plants conferring resistance to *H. zea* larvae attempting to enter the ear and feed on developing kernels (Cameron and Anderson, 1966; Wiseman and Davis, 1990). Plant structures may also act to hide the herbivore from its natural enemies. For example, open-leaf brassica varieties, such as Brussels sprouts, have higher parasitism on *P. rapae* compared to heading varieties, such as cabbage, due to larvae being able to feed in leaf folds protected from parasitoids (Pimentel, 1961). Furthermore, the size of plant structures impacts the ability of parasitoids to oviposit in pests, particularly if larger fruits allow pests to feed deeper than the parasitoid’s ovipositor can reach, creating “enemy-free space” and potentially facilitating host switching by pests (Bush, 1974; Price et al., 1980; Jeffries and Lawton, 1984; Bernays and Graham, 1988).

The plant surface is a complex microenvironment playing a critical role in insect–plant interactions, impacting insect behavior (such as attraction, retention, and host choice), feeding (such as attachment and accessibility of nutrients), and dispersal (by impeding insect movement) (Chapman, 1977; Southwood, 1986). Leaf surface structures that defend the plant from herbivores, such as leaf toughness, cuticle thickness, epicuticular waxes, trichomes and spines, can have direct and indirect effects on natural enemies. An indirect effect can occur if physical defense traits, such as leaf toughness, delay the development of herbivores. The extended period of vulnerability to natural enemies can thereby enhance biological control (slow-growth–high-mortality hypothesis, see Plant Nutrient-Mediated Interactions). A common example of direct effects is when trichomes are physically disruptive to natural enemy movement. In general, trichomes have more harmful than beneficial effects on predators, although most of these effects are sublethal (Riddick and Simmons, 2014a,b). The functional response or attack rate of predators and parasitoids is typically lower when their prey or hosts are found on plants with greater trichome density (e.g., Krips et al., 1999; Kumar et al., 1999; De Clercq et al., 2000; Stavrinides and Skirvin, 2003; Madadi et al., 2007; Jalalizand et al., 2012), although the opposite has been found as well (Koveos and Broufas, 2000). These interactions have significant implications for pest management; for example, biological control is possible on glabrous cucumber varieties, but is seriously hindered on those with dense trichomes due to the reduction in searching efficiency by the parasitoid *Encarsia formosa* Gahan (Hymenoptera: Aphelinidae) attacking greenhouse whiteflies *Trialeurodes vaporariorum* Westwood (Hemiptera: Aleyrodidae) (Hulspas-Jordaan and van Lenteren, 1978). Clusters of trichomes on the underside of plant leaves can form domatia, commonly used by predatory arthropods for shelter (O’Dowd and Willson, 1991; Walter, 1996; Agrawal and Karban, 1997); the positive impact of domatia on biological control has been well-documented for predatory phytoseiid mites (reviewed in Schmidt, 2014). In general, arthropods need to be either quite large (Rabb and Bradley, 1968; Obrycki and Tauber, 1984) or very small (Krips et al., 1999) to move along a leaf surface unimpeded by physical plant defense structures. The effect of trichome density on natural enemy movement can be a function of the relationship between natural enemy size and trichome spacing (Buitenhuis et al., 2014).

This myriad of physical plant traits clearly has an important effect on the feeding efficiency of herbivores. However, integration of plant physical traits with biological control is a complex issue with characteristics hindering herbivore damage also affecting (positively and negatively) the ability of natural enemies to attack pest species. This trade-off is evident in many examples of physically mediated interactions. In addition to trichomes, another plant surface characteristic that can impact natural enemies is the presence and composition of epicuticular waxes, which will be discussed in the following section.

#### Case Study: Plant Epicuticular Waxes, the Diamondback Moth, and Its Predators

Plant epicuticular waxes primarily serve to control water, gas and solute exchange (Riederer and Müller, 2006). In addition, these waxes mediate other ecological functions including host plant resistance against pathogens (Reina-Pinto and Yephremov, 2009) and herbivores (Eigenbrode et al., 1991b; Müller, 2008). The interactions between *B. oleracea* (cabbage, broccoli, cauliflower, kale, and others), *Plutella xylostella* (L.) (Lepidoptera: Plutellidae), and its predators highlight the interface between plant waxes and herbivore resistance. Gene mutations yield *B. oleracea* cultivars with altered chemical structures and different crystallization patterns of epicuticular lipids (Macey and Barber, 1970; Netting et al., 1972; Baker, 1974). As a consequence, mutants usually have decreased epicuticular waxes and produce a “glossy” phenotype instead of their normal wax “glaucous” phenotype (Eigenbrode and Espelie, 1995). Although information is limited (Verkerk and Wright, 1996), evidence suggests that glossy plants exhibit resistance against neonate *P. xylostella* larvae (Lin et al., 1983; Eigenbrode and Shelton, 1990; Eigenbrode et al., 1991a) and that physical and chemical differences influence neonate behavior (Eigenbrode et al., 1991b). Neonates on glossy varieties disperse further and faster, spending less time palpating, biting, mining, and spinning silk (Eigenbrode and Shelton, 1990; Eigenbrode et al., 1991a). This non-preference behavior causes a lack of establishment, reduced feeding and increased larval mortality (Eigenbrode and Shelton, 1990; Eigenbrode et al., 1991a).

Host plant resistance conferred by the glossy phenotype is also enhanced by predators. Field studies revealed that green lacewing *Chrysoperla carnea* (Stephens) (Neuroptera: Chrysopidae), insidious flower bug *Orius insidiosus* (Say) (Hemiptera: Anthocoridae), and convergent lady beetle *Hippodamia convergens* Guérin-Méneville (Coleoptera: Coccinellidae), all generalist predators, significantly increased *P. xylostella* larval mortality in glossy, but not normal wax, varieties (Eigenbrode et al., 1995). The reduction in mining behavior renders the larvae more exposed to predators (Eigenbrode et al., 1995). Predators also walked faster, spent more time walking, and covered more leaf area on glossy leaves compared to normal wax varieties (Eigenbrode et al., 1996). Increased mobility was attributed to increased traction/adhesion of predators on glossy vs. normal wax plants. The crystallization and composition of natural waxes have an impact on how natural enemies, such as *H. convergens* and *Chrysoperla plorabunda* (Fitch) (Neuroptera: Chrysopidae) attach to the leaf surface, thereby affecting their ability to exert biological control (Eigenbrode et al., 1999; Eigenbrode and Jetter, 2002).

In summary, this system has multiple pest suppression factors working together. *Plutella xylostella* neonates are less likely to accept glossy varieties, which increases their mortality and vulnerability to predation (via decreased mining behavior). Predators on glossy varieties have a greater ability to walk and hence, locate and attack prey, due to increased adhesion to the surface of leaves. Altogether, host plant resistance for *P. xylostella* in glossy varieties increases biological control by natural enemies, and hence overall suppression of this key pest of *Brassica* plants.

### Mechanisms of Plant Trait-Mediated Interactions: Summary

Plant traits have a profound (and often complex) array of impacts on herbivores and natural enemies. The examples cited within each section above for semiochemically, plant toxin-, plant nutrient-, and physically mediated interactions show the diversity and gradient of interactions occurring between natural enemies and HPR and how these can interact synergistically or antagonistically to suppress the target pest. For instance, semiochemically mediated traits serve as indirect plant defenses by impacting signaling pathways and attraction/repellency between the members of tritrophic interactions. Conversely, plant toxins act as direct defense against herbivores and this in turn can alter host suitability for natural enemies. Insect host/prey vulnerability via the slow-growth–high-mortality hypothesis can be mediated by plant nutrition. Plant-provided nutritional resources can also be linked to the success of natural enemies due to omnivory by predators and/or parasitoids. Moreover, physically mediated traits are known to function together with other traits to deter herbivory, but physical plant defenses are also responsible for increasing or decreasing herbivores’ vulnerability to natural enemies and trichomes can have direct negative impacts on biological control by decreasing natural enemy search efficiency. Manipulation of plant traits through plant breeding or bioengineering, as well as knowledge of the ecology and biology of herbivores and natural enemies, can work together to aid crop protection. In the last two decades, another control tactic, Bt, has become a staple of the agricultural landscape throughout much of the world (although notably less so in Europe). This technology will be discussed below given its importance in pest control programs throughout the world.

## Genetically Modified Crops and Interactions with Biological Control

Transgenic genetically modified (GM) crops have been engineered to incorporate genes derived from another species that confer nutritional or agronomic benefits, such as resistance to insect pests, viruses, herbicides, or protection from environmental conditions (e.g., low water availability). Among insect-resistant GM crops, *Bacillus thuringiensis* (Bt) crops are the most common and express insecticidal proteins derived from a naturally occurring soil bacterium. The insecticidal mode of action occurs when Bt toxins bind to receptors on the midgut lining of susceptible insects, causing lysis of epithelial cells on the gut wall, perforations in the midgut lining, cessation of feeding, and death by septicemia. Bt toxins target a narrow spectrum of pest insects that possess specific physiological traits (i.e., gut pH and toxin receptor sites in the midgut), and thus pose less direct toxicity risk to non-target species than broad-spectrum insecticides (Marvier et al., 2007; Wolfenbarger et al., 2008; Naranjo, 2009; Duan et al., 2010; Peterson et al., 2011). Commercialized Bt crops include maize, cotton, and soybeans that are protected against a suite of coleopteran and lepidopteran pests. The planting of Bt crops has increased dramatically since their introduction in the mid-1990’s; for example, in the United States, the percentage of Bt maize was only 1% of the total crop grown in 1996 but 81% of all maize grown in 2015 (United States Department of Agriculture National Agricultural Statistics Service, 2015). The ecological interactions between insect-resistant GM crops and biological control are complex and have been addressed in numerous comprehensive reviews (e.g., Obrycki et al., 2004; Lundgren et al., 2009a; Hilbeck and Otto, 2015). Two major categories for how GM crops influence biological control, proposed by Lundgren et al. (2009a), are discussed below: (1) toxicity-based pathways, including natural enemy consumption of toxic plant or prey foods; and (2) crop-induced changes to the environment, including unintended alterations to the crop plant and a decrease in prey quality and/or density that alter functional and numerical responses as well as the community ecology of natural enemies.

Many natural enemies consume plant-provided non-prey foods (see Plant Nutrient-Mediated Interactions) and when these plant-provided resources are GM crops, they are likely to contain Bt toxins. The expression of transgenic proteins is influenced by many biotic and abiotic factors, including environment, geography, crop phenology and genetics, and the specific transgenic event and protein expressed (Fearing et al., 1997; Duan et al., 2002; Grossi-de-Sa et al., 2006; Obrist et al., 2006a; Lundgren et al., 2009a). Most Bt crops employ a constitutive promoter that expresses Bt proteins throughout the life of the plant in nearly all tissues. Natural enemies that engage in facultative phytophagy of these plants are therefore likely to be exposed to the Bt toxins. Despite this exposure, laboratory feeding assays and field studies do not report negative impacts (Pilcher et al., 1997; Armer et al., 2000; Lundgren and Wiedenmann, 2002; Geng et al., 2006; Ludy and Lang, 2006; Obrist et al., 2006b; Torres et al., 2006; Li et al., 2008), most likely due to the high specificity of Bt proteins against target pests and the lack of necessary physiological conditions in non-target arthropods. It is therefore unlikely this pathway has a significant impact on biological control in transgenic crops.

Natural enemies may be exposed to Bt toxins by consuming or parasitizing prey/hosts that have fed on GM crops, a pathway similar to plant toxin-mediated interactions (see Plant Toxin-Mediated Interactions). One factor mitigating the exposure of natural enemies is that for crop pests that are highly susceptible to Bt toxins, ingestion of a very small amount of toxin elicits lethal effects. Exposure to natural enemies can be greater if the herbivore consuming a GM crop plant is only partially susceptible to the toxin and therefore consumes a greater quantity of plant tissue. Many herbivores do contain transgenic toxins (e.g., Harwood et al., 2005; Meissle et al., 2005; Obrist et al., 2005, 2006b; Peterson et al., 2016), but accumulation in higher trophic levels is uncommon (Dutton et al., 2002; Obrist et al., 2006a; Paula and Andow, 2016). While tritrophic transfer of Bt proteins has been documented, it is at low levels (e.g., Harwood et al., 2005, 2007; Meissle et al., 2005; Zwahlen and Andow, 2005; Obrist et al., 2006a; Wei et al., 2008; Chen et al., 2009; Meissle and Romeis, 2009; Peterson et al., 2009, 2016; Tian et al., 2010; Han et al., 2015). Early studies reported that some predators had negative sub-lethal effects from exposure to Bt-containing prey (Hilbeck et al., 1998a,b; Ponsard et al., 2002) but it was subsequently revealed that this was the result of reduced prey quality rather than direct exposure to Bt toxins (Romeis et al., 2004; Torres and Ruberson, 2006).

The most likely action by which GM crops could influence natural enemy fitness and fecundity is through a reduction in prey quality and/or prey density. Numerous studies have shown that consumption of Bt-containing plant tissue negatively affects the growth and development of herbivorous species, thereby impacting their natural enemies (e.g., Lövei and Arpaia, 2005; Hilbeck and Schmidt, 2006; Romeis et al., 2006; Lawo et al., 2010; Garcia et al., 2012; Tian et al., 2014; Han et al., 2015). For example, Hilbeck et al. (1998a) reported that the generalist predator *C. carnea* experienced reduced larval survival and longer development time when fed a diet of European corn borer (ECB), *Ostrinia nubilalis* (Hübner) (Lepidoptera: Crambidae), that had consumed Bt corn. However, generalist predators are capable of preferential feeding on healthy prey (Ferry et al., 2006) and are able to shift their dietary preferences to consume the mixture of nutrients required for optimal fitness (Mayntz et al., 2005; Raubenheimer et al., 2007; Marques et al., 2015). Therefore, generalist predators may be able to compensate for reduced quality of select prey due to Bt toxin consumption, having a negligible impact on biological control. For entomopathogens, species that are specialists of Bt-targeted pests are likely to see population reductions, whereas generalists will continue to persist in Bt crop fields (Obrycki et al., 2004). Parasitoids often do not have the flexibility to select hosts unaffected by Bt toxins and are therefore more likely to be adversely affected (Bernal et al., 2004; Marvier et al., 2007; Wolfenbarger et al., 2008; Bernal, 2010). Specialist parasitoid populations are reduced due to a lack of suitable hosts and may also suffer direct mortality if they are developing inside of a host that suffers mortality due to ingestion of Bt toxins (Agrawal, 2000a). For hosts that are only partially susceptible to Bt toxins, reduced host quality can result in sublethal effects on parasitoids (e.g., Bernal et al., 2002; Baur and Boethel, 2003; Vojtech et al., 2005; Ramirez-Romero et al., 2007; Walker et al., 2007), but host-mediated impacts of Bt crops on parasitoids are not universal and vary depending on the plant, host, and parasitoid. For example, the soybean looper *Chrysodeixis includens* (Walker) (Lepidoptera: Noctuidae) is moderately susceptible to the Bt toxins expressed in transgenic cotton and exhibits slower development time and lower prepupal weight (Baur and Boethel, 2003). Parasitism by *Cotesia marginiventris* (Cresson) (Hymenoptera: Braconidae) on these hosts results in longer larval development time, reduced adult longevity, and reduced egg production. However, when *C. floridanum* parasitizes loopers that have fed on Bt cotton, wasp pupal development time and adult longevity are unaffected, but fewer adults are produced per host (Baur and Boethel, 2003), revealing the difference in effects between species. In addition to development time, natural enemy size can be reduced if feeding on lower quality prey or hosts; smaller size in insects can result in reduced fecundity and dispersal capacity (Honěk, 1993; Kazmer and Luck, 1995), further delaying natural enemy population growth (Lundgren et al., 2009a).

The majority of interactions discussed above operate at the scale of a single crop field or smaller. However, some effects of the proliferation of GM crops are observed at the landscape or community scale. For example, Bt maize has been associated with area wide suppression of ECB in the midwestern United States (Hutchison et al., 2010). Despite reduced ECB populations that confer economic benefits to growers planting non-Bt maize, management of this pest is still critical for seed corn, popcorn, and other crops not protected by Bt toxins. Therefore, suppression of ECB due to biological control by natural enemies such as the specialist parasitoid *Macrocentrus grandii* (Goidanich) (Hymenoptera: Braconidae) and the entomopathogenic microsporidian *Nosema pyrausta* (Paillot) (Microsporidia: Nosematidae) is a valuable service. Despite the large reduction in ECB populations, infection dynamics of *N. pyrausta* have not significantly changed (Lewis et al., 2009), although parasitism rates by *M. grandii* were lowest when ECB hosts were found in small aggregations (White and Andow, 2005). Therefore, the area wide suppression of Bt-targeted prey or hosts does not always affect the interactions of pests with their natural enemies.

In addition to transgenic Bt crops, other herbicide-resistant and insecticidal GM crops are commercially available or under review by governmental agencies. The adoption of herbicide-tolerant crops that confer resistance to herbicides such as glyphosate, glufosinate, and 2,4-Dichlorophenoxyacetic acid (2,4-D) has been rapid. In the United States, 89% of corn and upland cotton and 94% of soybeans planted in 2015 had GM herbicide-tolerance traits (United States Department of Agriculture National Agricultural Statistics Service, 2015). Furthermore, herbicide-tolerant canola, alfalfa, and sugar beets are currently being grown in the United States, albeit in reduced frequency. This adoption has led to changes in the agricultural landscape, including reduced within-field plant diversity (Heard et al., 2005; Culpepper, 2006; Pleasants and Oberhauser, 2013), potentially affecting natural enemies and conservation biological control. The potential consequence of GM herbicide-tolerant crops on biological control is addressed in detail by Lundgren et al. (2009a). Transgenic insecticidal traits other than Bt have been studied; for example, potatoes, rice, maize, sugarcane, wheat, and other crops have been engineered to express snowdrop lectin GNA, a protein produced by the common snowdrop plant *Galanthus nivalis* (Asparagales: Amaryllidaceae) that expresses anti-hemipteran properties (Gatehouse et al., 1996; Sudhakar et al., 1998; Wang et al., 2005; Zhangsun et al., 2007; Duan et al., 2015). However, negative impacts of snowdrop lectin on natural enemies have been reported (Birch et al., 1999; Sétamou et al., 2002a,b,c; Horgervorst et al., 2006; Li and Romeis, 2009). The next generation of transgenic insecticidal crops in the commercial pipeline utilizes RNA interference (RNAi), where small double stranded RNA molecules expressed in the plant selectively silence targeted genes in herbivores that feed on the plant (Siomi and Siomi, 2009). For the western corn rootworm (WCR), silencing the *DvSnf7* gene using genetically modified RNAi maize induces mortality of this pest (Baum et al., 2007; Bolognesi et al., 2012) but the interactions between RNAi crops and biological control are not fully understood. While the reported spectrum of insecticidal activity of *DvSnf7* RNAi is limited to a subset of species related to the WCR (Bachman et al., 2013), further risk-assessment is clearly required. The potential hazards of GM RNAi crops to natural enemies include off-target gene silencing, silencing of the targeted gene in non-target organisms, immune stimulation, and saturation of the RNAi machinery; however, these interactions may be highly complex and difficult to predict (see reviews by Lundgren and Duan, 2013; Casacuberta et al., 2015; Roberts et al., 2015). Consequently, understanding the potential effect that GM crops have on natural enemy-pest dynamics will allow for better integration of this technology with biological control services. Genetically engineered biotech crops undoubtedly afford significant levels of pest suppression; research on the compatibility of this approach with biological control is critical to address the long-term integration of both approaches.

## Discussion

### Top-Down vs. Bottom-Up Control of Herbivorous Populations

As emphasized throughout this review, IPM ideally integrates a range of approaches to reduce damage caused by insect pests. Two of these approaches, HPR and biological control, are essentially forms of bottom-up and top-down control of herbivore populations. Whether breeding for increased plant resistance and the use of biological control are compatible and complementary approaches depends, in large part, on the mechanisms involved in HPR and the effects they have on biological control agents. Plant breeding for increased toxicity to herbivores will likely have negative effects on any biological control agents of these herbivores, whether due to direct ingestion of plant toxins or the effects of reduced host or prey size. In this respect, the array of interactions described in the Plant Toxin-Mediated Interactions and Plant Nutrient-Mediated Interactions sections are expected to apply here. An increasing number of studies have demonstrated that HPR has negative consequences for biological control agents through reduced body size or survivorship of individual natural enemies, raising the concern that such approaches are incompatible. Perhaps true in some circumstances, this is not always the case. Even if these control tactics negatively interact, the net effect in suppressing pest populations may be greater than use of either strategy alone. While rarely done, studies evaluating the joint effects of HPR and biological control efforts on pest population dynamics are essential to design effective and sustainable IPM strategies to minimize pest damage. Conversely, efforts to increase HPR by selecting for varieties that increase production of volatiles attractive to biological control agents are clearly compatible with biological control approaches. These interactions have been discussed in the Semiochemically Mediated Interactions and Case study: Maize Volatiles, Western Corn Rootworm, and Entomopathogenic Nematodes sections. Too often, however, little is known about the mechanisms underlying plant resistance to herbivory.

In turn, parasitoids can reduce herbivore pressure allowing for increased plant yields. Parasitoids, especially solitary species, can reduce damage done by herbivores, resulting in direct yield benefits to the plant; even gregarious parasitoids, which often induce increased feeding by individual herbivores, can reduce long-term population sizes of herbivores. Indeed, the widespread success of many insect biological control programs speaks to the ability of parasitoids (and predators) to have positive effects on plant production and yield. An underappreciated facet of this interaction between parasitoids and plant fitness/yield is the potential for parasitoids to reduce the likelihood of evolution of herbivore resistance to plant resistance traits. This is discussed further in section “Biological Control Can Reduce the Likelihood of Resistance Evolution.”

### Considerations for the Use of Volatiles to Recruit Biological Control Agents

Most studies involving HIPVs are undertaken in laboratory and greenhouse settings, with fewer studies conducted on the efficacy of HIPVs as host–plant resistance mechanisms in cropping systems at the field scale (Orre et al., 2010; Simpson et al., 2011a,b). Our understanding of arthropod responses to chemical compounds is still evolving, but efforts in developing HIPV strategies for crops are already in place via baiting/lures (Kaplan, 2012) or via bioengineering (Degenhardt et al., 2003, 2009). However, efforts to increase natural enemy efficacy by increasing plant attractiveness via HIPVs cannot ignore potential side effects. Extensive reviews of the challenges and the future of HIPV use in pest management have been published (Dicke, 2009, 2015; Alba et al., 2012; Kaplan, 2012; Heil, 2014) and there are many unknown factors and risks associated with the use of HIPV-based pest management tactics. Cropping systems are often considered low-diversity environments because of monocultural practices but in reality there are a multitude of organisms in any given field emitting and receiving chemical cues. We know that HIPVs targeted to attract natural enemies also attract herbivores, plant parasites, and members of the fourth trophic level. Releasing HIPV technology without examining the ecological factors present may render the technology ineffective. Several studies have shown that application of synthetic elicitors such as methyl jasmonate (MeJA) to induce elevated plant volatile production can also attract herbivores (Ballhorn et al., 2013) as well as hyperparasitoids (Kaplan, 2012; Heil, 2014), both outcomes that would be counterproductive to the potential for increased rates of parasitism by primary parasitoids. Additional spatio-temporal considerations must be understood to apply this technology in a large field setting. Moreover, it is unclear how the intentional use of HIPV technology impacts the net-efficiency of the HIPV-emitting crop. For example, the use of synthetic green leaf volatiles and MeJA to induce increased HIPV production in field grown maize did not result in increased parasitism rates by parasitoids of *S. frugiperda* (von Mérey et al., 2011, 2012). An essential question that needs additional exploration is whether an increase in biological control due to HIPV-emission will equate to increased crop yields.

### Biological Control Can Reduce the Likelihood of Resistance Evolution

Pesticide resistance is listed as the third most serious threat to global agriculture (behind soil erosion and water pollution) (Pimentel, 2005). Resistance is a pest population’s decreased response to a pesticide or control agent (including plant defense traits) as a result of previous exposure (McKenzie, 1996) and over 540 arthropod species have developed resistance to at least one pesticide (Arthropod Pesticide Resistance Database, 2016). The evolution of resistance to GM crops is of particular concern. For example, the WCR developed resistance to Cry3Bb1 Bt proteins with cross-resistance to mCry3A within 8 years of commercial release in the U.S. (Gassmann et al., 2011; Wangila et al., 2015). The impacts of resistance are often severe and far-reaching: they can lead to economic losses and increased pesticide usage. Delaying or preventing adaptation to pesticides, insecticidal GM crops and host plant defense traits can be achieved through the adoption of an integrated resistance management plan, and biological control can play a large role in these efforts. The impact of biological control on the rate of evolution of pest resistance is dependent upon whether natural enemies disproportionately attack resistant prey/hosts (thereby slowing resistance evolution) or susceptible prey/hosts (thereby accelerating resistance evolution) (Gould et al., 1991). In a high-dose/refuge strategy, such as that used for Bt crops, susceptible pests developing in refuges are frequently found at higher densities than resistant pests feeding on high-dose plants. Therefore, if natural enemies preferentially attack hosts found at higher densities (positive density-dependent mortality), the rate of resistance evolution will be faster than if natural enemies prefer less dense hosts (inverse density-dependent mortality) or are unaffected by host density (density-independent mortality) (Heimpel et al., 2005). For example, *Coleomegilla maculata* De Geer (Coleoptera: Coccinellidae) exhibits inverse density-dependent predation on the egg masses of the Colorado potato beetle *Leptinotarsa decemlineata* Say (Coleoptera: Chrysomelidae), decreasing the rate at which this pest develops resistance to Bt potatoes (Arpaia et al., 1997). However, the introduction of alternative prey can alter feeding patterns of this generalist predator, thereby affecting its influence on resistance evolution (Mallampalli et al., 2005).

Natural enemies can enhance resistance management for plant defense traits by inflicting mortality on those pests that have developed resistance (Liu et al., 2014). In oilseed rape *Brassica napus* L. (Brassicales: Brassicaceae) expressing Bt toxins, for example, the parasitoid *Cotesia vestalis* (Halliday) (Hymenoptera: Braconidae) dies with their host if developing inside a Bt-susceptible *P. xylostella* larva, but does not suffer negative effects when parasitizing Bt-resistant caterpillars (Schuler et al., 1999). Susceptible *P. xylostella* are killed within 5 days of feeding on Bt plants and consumption of Bt leaves is significantly reduced for susceptible larvae than resistant larvae. Consequently, the parasitoid *C. plutellae* is more attracted to Bt-resistant hosts, as plants with greater feeding damage release more HIPVs, which are attractive to the parasitoid (Schuler et al., 1999). Additionally, natural enemies can slow the evolution of resistance if they increase the fitness costs associated with resistance to crop traits (Raymond et al., 2007) but alternatively may amplify selection for resistance if they attack susceptible prey or hosts more frequently (Gould et al., 1991). For example, susceptible *H. virescens* feeding on Bt tobacco *Nicotiana tabacum* L. (Solanales: Solanaceae) took longer to develop, exposing them to greater parasitism by *Campoletis sonorensis*, and had higher movement rates, increasing risk of infection by the entomopathogenic fungus *Nomuraea rileyi* (Farlow) Samson (Johnson and Gould, 1992; Johnson et al., 1997a,b).

As described, biological control can influence the rate of resistance evolution via top-down influence. However, the manner in which host plant resistance traits are implemented can also have an effect on the evolution of resistance through bottom-up selection. The durability of plant resistance traits is affected by a multitude of factors that influence selection pressure on herbivorous pests, such as planting of a monoculture of resistant plants vs. mixtures or refuges of non-resistant plants, the mechanism and efficacy of the resistance traits, and the use of pyramiding multiple resistance traits (Stout, 2013). To achieve the greatest durability of plant defense traits, and therefore a more stable and sustainable pest management strategy, both top-down and bottom-up methods for delaying evolution of resistance by arthropod pests should be employed.

### How Can We Integrate Host Plant Resistance and Biological Control?

Historically, developers of HPR and biological control programs have worked independently, seeking to find “single-solution approaches to pest problems” (Thomas and Waage, 1996). Communication between such disparate groups such as plant breeders and natural enemy ecologists may not be inherently high. In reality, there are at least four distinct groups that should come together to better integrate plant defense traits and biological control: (1) HPR researchers (including plant breeders), (2) biological control researchers, (3) ecologists studying community and tritrophic interactions, and (4) extension professionals who are implementing IPM programs and working directly with producers and their advisors (Thomas and Waage, 1996). How can these fields and groups be brought together? Currently, plant breeding for HPR includes the selection of plant traits with the goal of enhancing direct defenses against herbivorous pests, with little consideration for enhancing plant traits that could improve indirect defenses through the action of natural enemies against pests (Cortesero et al., 2000). Evaluating the impacts of plant resistance characteristics on common natural enemies in the assessment of plant varieties during breeding for HPR would aid in bringing these two methods together. Additionally, fundamental ecological literature and applied host plant resistance literature have suffered from a lack of integration, an observation that has persisted for nearly 30 years (Kogan, 1986; Stout, 2013). An adherence by the host plant resistance community to the three traditional categories of resistance: antibiosis, antixenosis and tolerance (Painter, 1951) may also account for the lack of consideration of the third trophic level (Stout, 2013). Induced, indirect host plant resistance, such as what is seen when herbivore feeding or oviposition on plants triggers the attraction of natural enemies, does not fit into the three traditional categories proposed by Painter (1951). To further our understanding of the interactions between plant defense traits and biological control, experts that can conduct research using natural history, molecular and genetic tools, and field experimentation must be brought together (Agrawal, 2000a).

### Practical Implementation of Host Plant Resistance and Biological Control in Integrated Pest Management

A successful IPM plan must account for the ecology and biology of the targeted pest(s), environmental factors, and agricultural management. It must be localized; a one size fits all approach will never be effective, yet area wide suppression programs encompassing large regions are sometimes necessary (Schellhorn et al., 2015). This is a significant challenge in making prescriptions. An HPR-biological control combination targeting the same pest may work in one region, but not another. Similarly, this combination may work for one type of pest, but not another, even within the same field. While HPR and biological control are two of the key pillars of IPM, other essential management tactics include cultural control and chemical control. Another key management tactic is the “stimulo-deterrent diversion” or “push-pull” strategy. Host plant resistance traits can contribute to the “push” component, while biological control by natural enemies may be enhanced by concentration of pests due to the “pull” component (Eigenbrode et al., 2016). Finding a compromise between the strategies of host plant resistance and biological control may prove to be advantageous for selecting management strategies that maximize pest suppression and minimize the likelihood of resistance by reducing selection pressure on pests. For example, glandular pubescence was bred into commercial potato clones for defense against aphids and leafhoppers (Tingey, 1982). In the absence of natural enemies, aphid populations are the lowest on plants with high trichome density; however, when natural enemies are present, biological control is greatest on plants with intermediate trichome density (Obrycki et al., 1983). Therefore, plants with intermediate trichome density were recommended for potato IPM due to their partial resistance to aphids, compatibility with natural enemies, and reduced risk for development of pest resistance (Obrycki et al., 1983). The concept of pairing a partially resistant crop plant with biological control was proposed by van Emden (1988) as two of the three components of a “pest management triad” for aphid control (the third being use of selective insecticides to cause mortality of pests but not natural enemies). Cortesero et al. (2000) identified leaf domatia, trichomes (in intermediate density), plant signaling via volatiles, and extrafloral nectaries as the most promising plant defense traits for positive synergy with biological control.

Plants experience a wide range of biotic associations (both beneficial and antagonistic) above- and belowground that interact in complex ways (Bezemer and van Dam, 2005; van Dam and Heil, 2011). Herbivory and pathogen pressures experienced belowground can influence above ground interactions between plants, herbivores, and higher trophic levels (e.g., Soler et al., 2007, 2012). Approaches that use beneficial root associates such as arbuscular mycorrhizal fungi and rhizobacteria can not only increase root production and have benefits on yield and aboveground growth, they can stimulate aboveground defensive chemistry providing protection against aboveground herbivores (Gehring and Bennett, 2009; Orrell and Bennett, 2013).

Any recommendations that are given to maximize the compatibility of host plant resistance and biological control must also consider other important agronomic and practical factors, such as water availability and water use efficiency, fertilization and nutrient availability, weed management, and disease management. However, multiple goals can sometimes be achieved by the adoption of a single practice. For example, indirect host plant resistance, pathogen resistance, and biological control can be simultaneously supported in the case with leaf domatia on grape leaves: both predatory and fungivorous mites use these structures for protection and their presence can decrease incidence of arthropod pests and powdery mildew, a major disease of grapes (Agrawal, 2000a; Norton et al., 2000). For crop producers, agronomic traits other than insect resistance, and ultimately yield, will be the deciding factors for variety or hybrid selection. For crops where the seed market is dominated by transgenics, there may be less choice for the farmer; often only the highest yielding hybrids are chosen for transformation; in order to have the Bt or herbicide resistance traits desired, a smaller pool of varieties are available. Plant breeding often focuses on enhancing agronomic traits, such as drought tolerance, with higher yields as a major driving factor. Therefore, breeding for resistance to arthropod pests may not be the highest priority. Many plant defense traits have been inadvertently lost or weakened through domestication and selective breeding to enhance yields (Brattsen, 1991; Loughrin et al., 1995; Pickett et al., 1997; Rasmann et al., 2005; Chen et al., 2015a,b). Often, indirect defenses that rely upon the attraction or provisioning of natural enemies have also been lost, although efforts have been made to restore these plant traits, such as EβC-production due to an oregano transgene in maize to attract nematodes to attack rootworm larvae (see Case study: Maize Volatiles, Western Corn Rootworm, and Entomopathogenic Nematodes) or artificial domatia added to commercial cotton plants, which increased the abundance of certain predators (Agrawal et al., 2000). Wild relatives of cotton do have leaf domatia (Fryxell, 1978) and molecular mapping has been used to identify the genes that affect pubescence in cotton (Wright et al., 1999), allowing for the selective expression of pubescence at the leaf vein axils (domatia) that could positively affect natural enemies and biological control in cotton. Looking back to wild relatives of domesticated plant species could be informative for discovering plant defense traits capable of controlling pest species.

Host plant resistance and biological control are both well-suited for adoption in developing countries due to their low cost and lack of need for specialized equipment. The costs of HPR are often built into the price of seed (and may be a one-time expense if farmers can harvest and plant their own seeds subsequently). Biological control may be completely free, if natural control or conservation biological control is used. However, the use of entomopathogens may require application equipment. These biological control methods are in contrast to other types of management, such as chemical control, which may require the use of expensive equipment that is not accessible to farmers in developing countries. A review of these considerations can be found in Thomas and Waage (1996). Finally, HPR and biological control are compatible with the ecological intensification theory of agricultural production, which focuses on the conservation and promotion of biodiversity to support ecosystem services in cropland (Geertsema et al., 2016).

## Conclusion

In one of the first reviews to address the interactions between host plant resistance and biological control for pest management, Bergman and Tingey (1979) stated that “interactions between plant resistance and arthropod predators and parasites remain poorly known.” Since that time, a large body of literature has addressed this important question. However, we will need to continue to explore the dynamic interactions between host plant resistance and biological control as these tritrophic interactions are impacted by changing global conditions, such as climate. It is now clear that the mechanisms by which plant defense traits and natural enemies interact are complex and may be synergistic, disruptive, or anywhere on the continuum between. Each is clearly a powerful tool for suppressing herbivore populations and continued efforts to utilize these methods in IPM are essential for environmentally and economically sustainable global crop production. This review provided synthesis for the many facets of these interactions and encompassed the many critical implications these interactions have for agriculture today.

## Author Contributions

All authors listed, have made substantial, direct and intellectual contribution to the work, and approved it for publication.

## Conflict of Interest Statement

The authors declare that the research was conducted in the absence of any commercial or financial relationships that could be construed as a potential conflict of interest.

The reviewers JG and AB and handling Editor declared their shared affiliation, and the handling Editor states that the process nevertheless met the standards of a fair and objective review.
